# Combining Theory, Model, and Experiment to Explain How Intrinsic Theta Rhythms Are Generated in an *In Vitro* Whole Hippocampus Preparation without Oscillatory Inputs

**DOI:** 10.1523/ENEURO.0131-17.2017

**Published:** 2017-08-07

**Authors:** Katie A. Ferguson, Alexandra P. Chatzikalymniou, Frances K. Skinner

**Affiliations:** 1 Krembil Research Institute, University Health Network, Toronto, Ontario, Canada; 2 Department of Physiology, University of Toronto, Toronto, Ontario, Canada; 3 Departments of Medicine (Neurology) and Physiology, University of Toronto, Toronto, Ontario, Canada

**Keywords:** Computational modeling, hippocampus, mathematical model, network, theta rhythm

## Abstract

Scientists have observed local field potential theta rhythms (3–12 Hz) in the hippocampus for decades, but understanding the mechanisms underlying their generation is complicated by their diversity in pharmacological and frequency profiles. In addition, interactions with other brain structures and oscillatory drives to the hippocampus during distinct brain states has made it difficult to identify hippocampus-specific properties directly involved in theta generation. To overcome this, we develop cellular-based network models using a whole hippocampus *in vitro* preparation that spontaneously generates theta rhythms. Building on theoretical and computational analyses, we find that spike frequency adaptation and postinhibitory rebound constitute a basis for theta generation in large, minimally connected CA1 pyramidal (PYR) cell network models with fast-firing parvalbumin-positive (PV^+^) inhibitory cells. Sparse firing of PYR cells and large excitatory currents onto PV^+^ cells are present as in experiments. The particular theta frequency is more controlled by PYR-to-PV^+^ cell interactions rather than PV^+^-to-PYR cell interactions. We identify two scenarios by which theta rhythms can emerge, and they can be differentiated by the ratio of excitatory to inhibitory currents to PV^+^ cells, but not to PYR cells. Only one of the scenarios is consistent with data from the whole hippocampus preparation, which leads to the prediction that the connection probability from PV^+^ to PYR cells needs to be larger than from PYR to PV^+^ cells. Our models can serve as a platform on which to build and develop an understanding of *in vivo* theta generation.

## Significance Statement

Brain rhythms have been linked to cognition and are disrupted in disease. This makes it essential to understand mechanisms underlying their generation. Together with experiments, theory and mathematical models help provide an understanding and contribute to a framework to dissect out the cellular contributions to network activity. However, models are inherently biological approximations, and thus the specific experimental and theoretical context on which they are built will shape their output. If the approximations and contexts are not taken into account, particularly when using previously constructed models, misinterpretations can arise. Here we develop microcircuit models that are mapped onto specific experiments, allowing us to obtain essential balances for generation mechanisms of a dominant rhythm in the hippocampus: the theta rhythm.

## Introduction

The goals of mathematical modeling in neuroscience are many and varied. For any particular study, modeling goals need to be clear, as they guide our decisions during model development. Developed models can be used to generate new hypotheses and investigate interactions across different scales. In doing this, it is helpful to consider what is meant by an explanation. Aristotle’s doctrine of the four causes (Falcon, 2015)—material, formal, efficient and final, as described and interpreted in Brette (2013)—is useful. The efficient cause is what triggers the phenomenon to be explained; the material cause refers to the physical substrate of the phenomenon; the formal cause is the specific pattern (“balance”) responsible for the phenomenon; and the final cause is the function of the phenomenon. Typically, as stated by Brette (2013), theoretical approaches tend to focus on formal and final causes, and experimental approaches on material and efficient causes. Although all four causes may be needed to obtain a complete understanding, considering these causes can serve to clarify modeling goals and guide usage of developed models.

Electrical oscillations, as recorded in electroencephalograms and local field potentials (LFPs), are hallmarks of the brain that are linked to normal and pathologic functioning (Buzsaki, 2006). Thus, it is essential to understand the mechanisms underlying their generation. A large part of the challenge in obtaining mechanisms underlying oscillation generation is the multiscale nature of the brain, with its biological complexity and cellular specifics (Cohen and Gulbinaite, 2014). Various building blocks have long been known (Gjorgjieva et al., 2016), and it is clear that, for example, postinhibitory rebound building blocks contribute to the generation of cortical oscillations (McCormick et al., 2015). However, it is unclear if one can identify essential building block combinations and balances that underlie oscillation generation in the mammalian brain. In the Aristotelian sense, we have some insight into efficient causes (building blocks) for oscillation generation, but we do not yet have formal cause explanations.

Almost 80 years ago, Jung and Kornmuller discovered theta (3-12 Hz) rhythms in the rabbit (Colgin, 2013). These dominant rhythms are associated with memory processing and spatial navigation, present when the animal is actively exploring or during REM sleep. They can be parsed into atropine-resistant or atropine-sensitive types, with higher or lower theta frequencies, respectively (Buzsaki, 2002; Colgin, 2013, 2016), and *in vitro* models of theta rhythms have been developed (Gillies et al., 2002; Traub et al., 2004). Further, low or high theta rhythms were found to be elicited in rats with fearful or social stimuli respectively (Tendler and Wagner, 2015). In the human hippocampus, theta rhythms are linked to similar behaviors (Lega et al., 2012), although it may be the case that they are associated with a wider behavioral repertoire relative to rodents, as they are present without sensory input (Qasim and Jacobs, 2016). Theta rhythms are heavily studied, but with multiple forms, pharmacological sensitivities, and interactions between brain structures, it is challenging to have a clear understanding of their generation.

To explain how theta rhythms are generated, we need to have models that can be mapped onto experiments. As discussed by Colgin (2013), it is traditionally thought that the medial septum (MS) is critical for the generation of theta rhythms, since they are disrupted when the MS is lesioned or inactivated. Indeed, to understand theta rhythms, many studies have explored, characterized, and modeled the interactions between MS and hippocampus (e.g., Brazhnik and Fox 1999; Wang 2002; Borhegyi et al., 2004; Hajós et al., 2004; Kocsis and Li, 2004; Manseau et al., 2008; Varga et al., 2008; Hangya et al., 2009). However, the hippocampus can exhibit theta rhythms without the MS (Goutagny et al., 2009). Further, distinct inhibitory cell populations, such as parvalbumin-positive (PV^+^) cells, fire at unique phases of the theta rhythm and play important roles in their generation (Varga et al., 2014; Amilhon et al., 2015). Ultimately, to understand the varied functional roles of these dominant rhythms and how they are modulated and controlled, we need to include cellular aspects and be clear about the particular form of theta. From a mathematical modeling perspective, this reduces to deciding what “parameters, parameters, parameters” (Skinner, 2012) and values to use and how to represent the biological system, given that any mathematical model is an approximation of the biology.

In this article, we develop microcircuit models that are mapped to an *in vitro* whole hippocampus preparation that spontaneously expresses theta rhythms. We take advantage of theoretical insights and the ability to readily do thousands of network simulations with our developed mathematical models. We present an explanation for intrinsic CA1 theta generation that has elements of efficient, material, and formal causes. It involves building blocks of spike frequency adaptation and postinhibitory rebound in large pyramidal cell populations coupled with fast-firing PV^+^ cells, in which there is a larger connection probability from PV^+^ to pyramidal cells relative to the other way.

## Materials and Methods

Here we summarize our overall strategy, the experimental context of the whole hippocampus preparation, and our mathematical models and analyses. We also describe previous and motivating modeling work that the results are built on.

### Overall strategy

**Figure 1.**
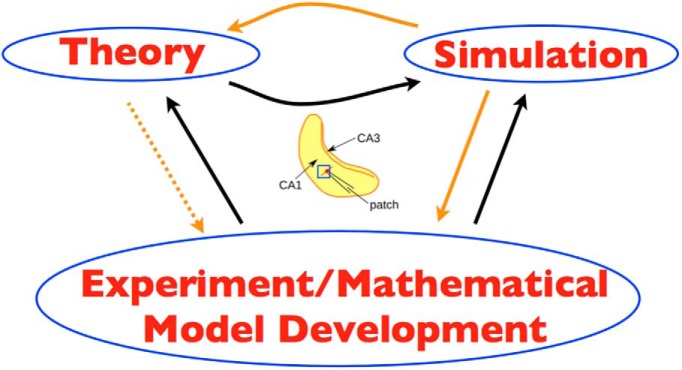
Overall strategy. The three schematic parts (left, right, lower) of theory, simulation, and experiment/mathematical model development are bidirectionally linked by arrows. Theory refers to mean field theory that was used to constrain the parameter sets to examine in simulations, using cellular models derived from experiment. Simulation refers to the computation of thousands of network simulations done. Experiment/mathematical model development refers to the cellular, Izhikevich-type models that were developed in the experimental context of the whole hippocampus preparation. In the middle is a schematic of the whole hippocampus preparation with an added blue square to illustrate the piece of tissue from the CA1 region of the hippocampus that is being modeled. The hippocampus schematic is adapted from Fig. 1 of Huh et al. (2016). Orange arrows are links in the present work and black arrows, in previous work. Dashed orange arrow from theory to experiment is because it is indirect, as theory previously contributed to simulation.

Our goal is to develop experimentally motivated microcircuit models of a hippocampal CA1 network to provide insight into the mechanisms underlying theta rhythm generation. Our approach is shown in the schematic of Fig. 1, where orange and black arrows refer to links in the present or previous work, respectively.

Cellular mathematical models of excitatory and inhibitory cells based on whole-cell patch clamp recordings from the whole hippocampus preparation were previously developed (Ferguson et al., 2013, 2015b). These cellular models were used to generate either excitatory (Ferguson et al., 2015a) or inhibitory (Ferguson et al., 2013) network models with sizes and connectivities as appropriate for the experimental context. A mean field theory (MFT) approach was taken advantage of to determine parameter regimes in the excitatory networks (Ferguson et al., 2015a).

In the present work, we combine these excitatory and inhibitory networks and perform a detailed computational analysis of this network. We investigate the dynamic interplay between these two cell populations and their roles in theta generation. Further theoretical analyses are required to fully understand the network dynamics. Overall, our strategy combines experiment, model development, simulation and theory. Our models bring together network size, connectivity, and cellular characteristics in a closed fashion given the experimental context.

### Experimental context

In 2009, Goutagny et al. (2009) developed an *in vitro* whole hippocampus rodent preparation that spontaneously generates theta (3–12 Hz) rhythms in the CA1 region. By blocking transmission across the septo-temporal axis, they identified multiple theta oscillators in the hippocampus (see Supplementary Fig. 11 in Goutagny et al. [2009]). Further, the presence of these theta oscillations was not dependent on the CA3 region (see Supplementary Fig.10 in Goutagny et al. [2009]) and required *GABA_A_* and *AMPA* receptors (see Supplementary Table 1 in Goutagny et al. [2009]). Given this, we estimate that the minimum circuitry required for CA1 theta rhythms is contained in ∼1 mm^3^. Using known cell densities and approximate volumes of axonal innervation (West et al., 1991; Aika et al., 1994; Sik et al., 1995; Jinno and Kosaka, 2006; Hosseini-Sharifabad and Nyengaard, 2007), we approximate that 30,000 excitatory pyramidal (PYR) cells and 500 PV^+^ cells are involved in the spontaneous generation of theta rhythms in the CA1 region of the hippocampus. This size estimate is thoroughly described in previous modeling work (Ferguson et al., 2013, 2015a).

**Table 1.** Cell model parameters

	PYR cell		PV^+^ cell
Parameter	Weakly adapting	Strongly adapting	
**v*_*r*_* (mV)	–61.8	–61.8	–60.6
**v*_*t*_* (mV)	–57.0	–57.0	–43.1
**v*_*peak*_* (mV)	22.6	22.6	–2.5
*a* (ms^–1^)	0.00008	0.0012	0.1
*b* (nS)	3	3	–0.1
*c* (mV)	–65.8	–65.8	–67
*d* (pA)	5	10	0.1
**k*_*low*_* (nS/mV)	0.5	0.1	1.7
**k*_*high*_* (nS/mV)	3.3	3.3	14
**C*_*m*_* (pF)	300	115	90
**I*_*shift*_* (pA)	–45	0	0

Subsequent work by Amilhon et al. (2015) indicates that networks of PV^+^ and PYR cells could encompass the basic (minimal) units required for theta rhythm generation in the whole hippocampus preparation. This is because optogenetically silencing PV^+^ cells eliminates the theta rhythm, whereas silencing somatostatin-positive inhibitory cells does not. Further, it is the case that PV^+^ cells receive large excitatory postsynaptic currents (EPSCs) relative to PYR cells during ongoing theta rhythms. Also, simultaneous recordings from PV^+^ or PYR cells with extracellular field recording of the theta rhythm indicates that the majority of PV^+^ cells fire phasically with the rhythm, whereas PYR cells fire sparsely (Huh et al., 2016). We estimate that 20% or less of the PYR cells are firing, based on Huh et al. (2016). Given that PV^+^ cells receive large EPSCs and that PYR cells fire sparsely, ongoing theta rhythms must necessarily be dependent on a large network effect.

During the ongoing theta rhythm, EPSCs in PYR cells are very small and variable (estimated to be <20 pA), whereas the inhibitory postsynaptic currents (IPSCs) to the PYR cells are larger (estimated to be ∼200 pA). Conversely, the PV^+^ cells receive very large EPSCs (estimated to be up to 1000 pA) and smaller IPSCs (∼200 pA). These estimates are based on whole-cell current recordings from Huh et al. (2016). Given these estimates, EPSC/IPSC ratios for PYR cells are <1, and EPSC/IPSC ratios for PV^+^ cells are >1.

Overall, we aim to determine the conditions under which our network models can produce population bursts at theta (3-12 Hz) frequency, given that there is sparse firing of excitatory PYR cells and non-sparse firing of inhibitory PV^+^ cells, and particular excitatory/inhibitory balances (Huh et al., 2016).

### Mathematical models

#### Cell model

Previously developed cellular models are based on experimental data from the *in vitro* whole hippocampus preparation (Ferguson et al., 2013, 2015b). They use the mathematical model structure developed by Izhikevich (2006, 2010), in which the subthreshold behavior and the upstroke of the action potential is captured and a reset mechanism to represent the spike’s fast downstroke is used. Despite being relatively simple, parameter choices can be made such that they have a well-defined (albeit limited) relationship to the electrophysiological recordings. The structure has a fast variable representing the membrane potential, *V* (mV), and a variable for the slow “recovery” current, *u* (pA). We used a slight modification to be able to reproduce the spike width. The model is given by

(1) $$\begin{array}{l} C_{m}\overset{˙}{V}=k(V-v_{r})(V-v_{t})-u+I_{shift}+I_{other}-I_{syn}\\ \overset{˙}{u}=a[b(V-v_{r})-u]\\ \text{if}\;V\geq v_{peak},\;\text{then}\;V\leftarrow c,\;u\leftarrow u+d\\ \text{where}\;k=k_{low}\;\text{if}\;V\leq v_{t},\;k=k_{high}\;\text{if}\;V>v_{t}, \end{array}$$

where *C_m_* (pF) is the membrane capacitance, *v_r_* (mV) is the resting membrane potential, *v_t_* (mV) is the instantaneous threshold potential, *v_peak_* (mV) is the spike cutoff value, *I_shift_* (pA) is a current that shifts the f–I curve laterally to allow the model to easily capture the rheobase current (for the strongly/weakly adapting models, rheobase current is ∼0/5 pA, respectively), *I_syn_* (pA) represents the synaptic input from the presynaptic cell population (further details below), *I_other_* (pA) is an (excitatory) current drive to the network that is not directly modeled through *I_syn_* (further details below), *a* (ms^–1^) is the recovery time constant of the adaptation current, *b* (nS) describes the sensitivity of the adaptation current to subthreshold fluctuations (greater values couple *V* and *u* more strongly, resulting in possible subthreshold oscillations and low-threshold spiking dynamics), *c* (mV) is the voltage reset value, *d* (pA) is the total amount of outward minus inward currents activated during the spike and affecting the after-spike behavior, and *k* (nS/mV) represents a scaling factor. Parameter values for the cell models (strongly and weakly adapting PYR and PV^+^ cell models) are given in Table 1.

#### Synaptic model

Synaptic input is modeled through a chemical synapse represented by

(2) $$I_{syn}=g\times s(V-E_{rev}),$$

where *g* (nS) is the maximal synaptic conductance of the synapse from a presynaptic neuron to the postsynaptic neuron, *E_rev_* (mV) is the reversal potential of the synapse, and *V* (mV) is the membrane potential of the postsynaptic cell. The gating variable, *s*, represents the fraction of open synaptic channels and is given by first-order kinetics (Destexhe et al., 1994; Ermentrout and Terman 2010, p.159):

(3) $$\overset{˙}{s}=\alpha [T](1-s)-\beta s.$$

The parameters α (in mM^–1^ms^–1^) and β (in ms^–1^) in Eq. 3 are related to the inverse of the rise and decay time constants (τ*_R_* and τ*_D_* in ms). [*T*] represents the concentration of transmitter released by a presynaptic spike. Suppose that the time of a spike is *t* = *t*_0_ and [*T*] is given by a square pulse of height 1 mM lasting for 1 ms (until *t*_1_). Then, we can represent

$$s(t-t_{0})=s_{\infty }+(s(t_{0})-s_{\infty })e^{[-\frac{t-t_{0}}{\tau_{s]}}},\;t_{0}<t<t_{1}$$

where

(4) $$\begin{array}{ccc} s_{\infty }=\frac{\alpha }{\alpha +\beta } & \text{and} & \tau_{s}=\frac{1}{\alpha +\beta }. \end{array}$$

After the pulse of transmitter has gone, *s*(*t*) decays as

(5) $$s(t)=s(t_{1})e^{-\beta (t-t_{1})}.$$

#### Network models

**Figure 2.**
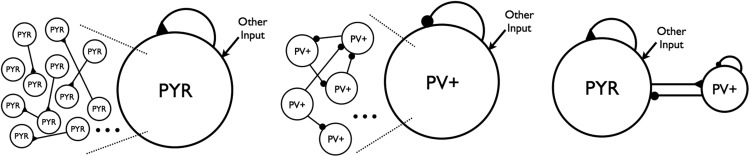
Network schematics. Left: Excitatory PYR cell networks. The large circle with PYR represents a population of individual PYR cells, and the black triangles represent excitatory connections. Middle: Inhibitory, fast-firing cell networks. The large circle with PV^+^ represents a population of individual inhibitory, fast-firing PV^+^ cells, and the black circles represent inhibitory connections. The connections are schematized so that there is clearly not all-to-all coupling. “Other” input is either deterministic or noisy, excitatory synaptic input. Right: Excitatory-inhibitory cell networks. PYR and PV^+^ cell populations combined to create networks of 10,500 cells. However, now the excitatory input to PV^+^ cells comes directly from the PYR cell network.

Excitatory, inhibitory, and excitatory-inhibitory network models are illustrated in Fig. 2 for PYR cell networks (top), PV^+^ cell networks (middle), and PYR-PV^+^ cell networks (bottom). Networks have either deterministic or noisy “other” input. If deterministic, then *I_other_* is a constant, tonic input to individual cells in the network, where *I_other_* is chosen from a normal distribution with mean *I_app_* (pA) and SD σ*_app_* (pA). If noisy, then $I_{other}=-g_{e}(t)(V-E_{rev})$. *g_e_*(*t*) is a stochastic process similar to the Ornstein–Uhlenbeck process as used by Destexhe et al. (2001):

(6) $$\frac{dg_{e}(t)}{dt}=-\frac{1}{\tau_{e}}(g_{e}(t)-g_{e,mean})+\sqrt{\frac{2\sigma_{e}^{2}}{\tau_{e}}}\chi_{e}(t),$$

where χ*_e_*(*t*) is an independent Gaussian white noise process of unit SD and zero mean, *g_e_*_,_*_mean_* (nS) is the average conductance, σ*_e_* (nS) is the noise SD value, and τ*_e_* is the time constant for excitatory synapses. τ*_e_* is fixed based on values as used in Destexhe et al.. (2001) (τ*_e_* = 2.73 ms).

#### Network model parameters, rationale

Random connectivity was used throughout, and the probability of connection is given in Table 2, where it is fixed for PYR or PV^+^ cell networks, as estimated in previous work. Network sizes and synaptic time constants are given in Table 2. Time constant values are taken from Papp (2013) for PYR-PV, Spruston et al. (1995) for PYR-PYR, and Bartos et al. (2002) for PV-PV and PV-PYR connections. Excitatory and inhibitory reversal potentials *E_exc_* and *E_inh_* as derived from the experimental context are –15 and –85 mV, respectively (Huh et al., 2016).

**Table 2.** Network model parameters

			Synaptic time constants	
Parameter	Number of cells	Probability of connection	Rise time (ms)	Decay time (ms)
PV^+^ cells	500			
PV-PV		0.12	0.27	1.7
PV-PYR		0.01 – 1	0.3	3.5
PYR cells	10,000			
PYR-PV		0.01 – 1	0.37	2.1
PYR-PYR		0.01	0.5	3

Connectivity between PV^+^ and PYR cells was not fixed, but ranged in values as indicated. A resolution of 0.01 was used from 0 to 0.1, and 0.1 upward.

#### PYR cell networks

*g_pyr_* (nS) is the maximal excitatory (AMPA) synaptic conductance between PYR cells. Parameter explorations for deterministic networks included: *I_app_* (pA) = [0, 5, 10,…, 75, 80] and σ*_app_* (pA) = [0, 5, 10, 15, 20]. Parameter explorations for noisy networks included: *g_e_*_,_*_mean_* (nS) = [0,1,2] (mainly, but values up to 10 explored) and σ*_e_* (nS) = [0,0.2,0.4,0.6]. *g_pyr_* (nS) = [0.014, 0.024, 0.034,…, 0.084, 0.094] was used in both deterministic and noisy networks.

#### PYR-PV^+^ cell networks

A full exploration was done for connectivity between PV^+^ and PYR cell networks (Table 2). We use *c_PV_*_,_*_PYR_* and *c_PYR_*_,_*_PV_* to refer to the probability of connection from PV^+^ to PYR cells or from PYR to PV^+^ cells, respectively. *g_pv_* is the maximal inhibitory synaptic conductance between PV^+^ cells, and a parameter value of 3 nS was used based on our previous PV^+^ cell network modeling (Ferguson et al., 2013).

##### Deterministic networks

Full connectivity explorations were done for chosen parameter sets: [*g_pyr_*,*I_app_*,*σ_app_*] = [0.014, 0, 0], [0.014, 0, 10], [0.024, 30, 0], [0.054, 5, 5], [0.054, 20, 20], and [0.074, 75, 15]. *g_pv_*_–_*_pyr_* (nS) is the maximal inhibitory synaptic conductance on PYR cells from PV^+^ cells. It was fixed at 8.7 nS, as approximated from IPSCs in Bartos et al. (2002), for most of the simulations. *g_pyr_*_–_*_pv_* (nS) is the maximal excitatory synaptic conductance on PV^+^ cells from PYR cells. A value of 1 nS was used for most of the simulations, as estimated from Papp (2013).

##### Noisy networks

Full connectivity explorations were done for *g_e_*_,_*_mean_* (nS) = [0, 1, 2], σ*_e_* (nS) = [0, 0.2, 0.4, 0.6] with *g_pyr_* = 0.014 nS, all *g_pyr_* values given above, and σ*_e_* = [0, 0.2, 0.4, 0.6] with *g_e_*_,_*_mean_* = 0 nS. *g_pv_*_-_*_pyr_* = 8.7 nS was used, but additional simulations using values of [6, 6.5, 7…., 11.5, 12] were done for two parameter sets with *g_e_*_,_*_mean_* = 0 nS; [σ*_e_*, *g_pyr_*, *c_PYR,PV_*, *c_PV,PYR_*] = [0.2, 0.084, 0.4, 0.5] and [0.6, 0.014, 0.02, 0.3]. Similarly, *g_pyr_*_-_*_pv_* = 3 nS was used and scaled for network size (see below). Additional simulations were done for *g_pyr_*_-_*_pv_* values of [0.5, 1, 1.5, 2,…, 5.5, 6] for the chosen parameter sets. Overall, close to 6000 simulations were performed.

### Analyses

For each network simulation, we defined the population activity as the average membrane potential of all model cells. Then, using the fast Fourier transform (fft), the network frequency (*f_peak_* in Hz) is defined as the frequency at which there is a spectral peak in the overall population activity. In this analysis, we disregard the initial transient activity (500 ms).

We defined a population burst based on the distribution of spikes of the PYR cell network. To do so, the total number of spikes within a small bin width were summed, where the bin width was dependent on the average peak frequency: bin width = $int(p_{1}\times round\{[(p_{2}\times exp(-p_{3}\times f_{peak}+p_{4})+p_{5}]/p_{1}\})$, where *p*_1_ = 2, *p*_2_ = 2.0264, *p*_3_ = 0.2656, *p*_4_ = 2.9288, and *p*_5_ = 5.7907, such that for peak frequencies ranging from *f_peak_* = 3–12 Hz, the bin width ranged from ≈23 to 7 ms. In this way, the bin would be smaller for higher frequencies. Then, the total number of spikes per bin width was normalized to its maximum (excluding transient activity within the first 500 ms) so that networks with significantly different levels of activity could be compared. A moving threshold capturing approximately five cycles was set to be the mean + 0.35 SDs of the local normalized distribution. Then, the burst was determined to be the midway point between the increase past threshold and the previous decrease past the threshold (with the requirement that these points are at least $1/(f_{peak}\times 2.5)\;ms$ apart). If the difference between the peak to trough of the burst is <0.2, it is no longer considered to be a burst. A population burst is considered to be more robust if the power of the fft is larger or the normalized size of the PYR cell spike distribution is larger. We note that if the population burst is reasonably robust, then the burst frequency as determined from the fft is essentially the same as the inverse of the burst width.

We automated the categorization of our network output for the different parameter sets explored. Specifically, nonfiring cases were considered when there were <300 spikes per burst bin. If network burst frequencies were within theta frequency ranges, they were further examined to determine their stability. Bursts were considered to be stable if there were at least two occurrences of two consecutive amplitudes decreasing by >79%. For each burst, we determined the burst width, the number of cells that fired in the burst, and the total number of spikes in the burst. In this way, we can not only track these properties for the network as a whole, but also determine how they change over time. This analysis was based on custom code written in Matlab.

For each simulation, we recorded EPSCs and IPSCs from 100 PYR and 50 PV^+^ cells and chose a subset to analyze. Specifically, we used peakfinder in Matlab and ignored any peaks that were below a value thresholded at an order of magnitude less than the main peaks. The first second was not included in the calculations. We computed averages and SDs of 3 PV^+^ and 5 PYR cells and rounded them to give the reported values.

Simulations were run using the Brian simulator (Goodman and Brette, 2009) on the GPC supercomputer at the SciNet High Performance Computing Consortium (Loken et al., 2010). The initial conditions of our membrane potentials (V) were chosen to be uniform random values from –55 to –65 mV. We used the forward Euler method for integration with a time step of 0.02 ms. PYR cell networks were simulated for 10 s, whereas PYR-PV^+^ cell networks were simulated for 4 s. For noisy simulations, simulations were done with second-order explicit Runge–Kutta numerical integration, with a time step of 0.04 ms. A subset of these simulations was also run with the forward Euler method and compared.

### Motivating modeling studies

#### Excitatory (PYR cell) networks, deterministic

In previous work, it was considered whether CA1 PYR cell networks on their own could generate theta rhythms (Ferguson et al., 2015a) or, more specifically, given CA1 PYR cell intrinsic properties, connectivity, and cell numbers, can one obtain theta frequency (3-12 Hz) population bursting as observed in the experimental context? This was directly addressed in Ferguson et al. (2015a). Individual PYR cell models were based on whole cell recordings from the whole hippocampus preparation (Ferguson et al., 2015b). Cells exhibited either weak or strong adaptation (determined by how much their frequency changed over the course of a 1 second long input), and postinhibitory rebound (spiking after being released from a hyperpolarizing current), and these models captured these properties. These PYR cell models were connected in a network (see left schematic of Fig. 2 and model details above) and MFT was used to find parameter regimes in which the network exhibited theta frequency population bursts (Ferguson et al., 2015a). Due to a scaling relationship between cell number, connection probability, and *g_pyr_* from the MFT, one can use 10,000, rather than 30,000, PYR cells in the network simulations.

Stable theta frequency population bursts emerged from these PYR cell network models without a phasic drive. Similar to what was predicted from the MFT simulations, larger mean excitatory drive, *I_app_*, was required to obtain theta frequency bursts as the SD of the drive, σ*_app_*, increased (Ferguson et al., 2015a). Burst frequencies were determined by three factors: the mean excitatory drive, *I_app_* (which increases the burst frequency as it increases); the recurrent synaptic strength, *g_pyr_* (which decreases burst frequency as it increases); and the SD of the excitatory drive across the cells, σ*_app_* (which increased frequencies as it increased) (Ferguson et al., 2015a; Ferguson, 2015).

**Figure 3.**
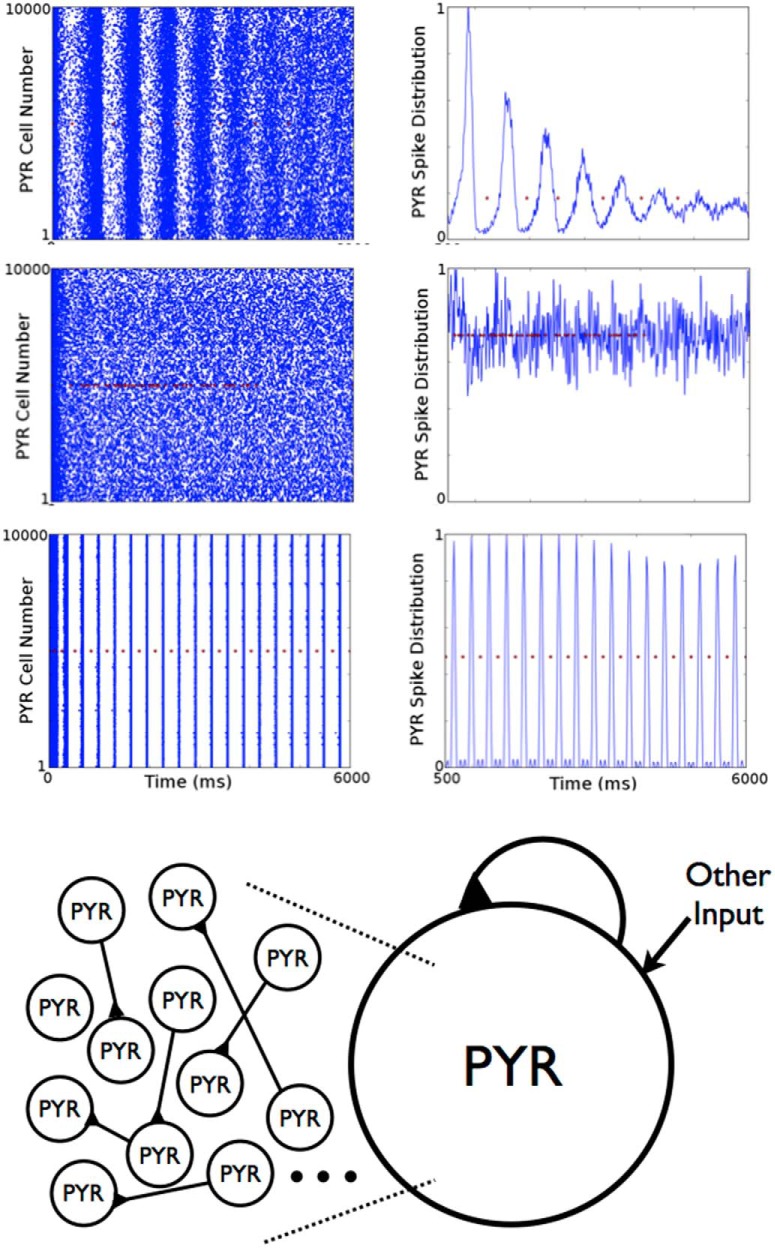
Deterministic excitatory PYR cell networks. Three examples are shown of raster plots and normalized spike distributions, one of which shows a theta frequency population burst. The spike distributions are used to determine the burst bins, and the red symbols represent the separation of the bins. *I_app_* and “other” input parameter values: *I_app_* = 5 pA, *g_pyr_* = 0.054 nS, σ*_app_* = 5 pA (top: unstable bursts); *I_app_* = 0 pA, *g_pyr_* = 0.014 nS, *I_app_* = 10 pA (middle: no bursts); *I_app_* = 30 pA, *g_pyr_* = 0.024 nS, *I_app_* = 0 pA (bottom: 3.1 Hz rhythm).

From the parameter sets explored, network output was automatically categorized (see specifics in above sections) such that nonfiring, stable theta frequency bursts, unstable bursts, or other groupings were apparent. In Fig. 3, we show three example outputs, one of which exhibits theta rhythms (bottom), another unstable bursts (top), and the third asynchronous behavior (middle). In the theta bursting parameter regimes, we analyzed our networks to determine how many PYR cells were firing (i.e., active) during the population bursts. We found that >90% of PYR cells are active when theta rhythms are present. In Table 3, we show the minimal number of active PYR cells for each σ*_app_* value. Except for σ*_app_* = 0, these minimal cases all have frequencies that are below theta but ∼50% of their 10,000 PYR cells are still active. Also, the number of active PYR cells during bursts increased with increasing *I_app_* and also with increasing *g_pyr_*. These observations are from simulations that were performed using strongly adapting PYR cell models. Weakly adapting PYR cell models were also used, but specifics are not shown; it is already known from previous MFT studies that more input drive is required to achieve theta frequency population bursts with the reduced cellular adaptation (Ferguson et al., 2015a).

**Table 3.** PYR cell networks: numbers of active, firing cells in population bursts

Parameter	Average number of active cells (/10,000) per population burst	Burst frequency (Hz) (estimated from fft)
Deterministic networks **g*_*pyr*_* (nS) **I*_*app*_* (pA), σ*_*app*_* (pA)		
[0.064, 65, 0]	9703	6.8
[0.024, 10, 5]	5541	1.4
[0.054, 10, 10]	4929	2.0
[0.014, 10, 15]	5075	1.2
[0.014, 10, 20]	5085	1.1
Noisy networks **g*_*pyr*_* (nS) **g*_*e,mean*_* (nS), σ*_*e*_* (nS)		
[0.014, 1, 0.6]	5992	5.2
[0.024, 1, 0.6]	6173	5.2
[0.034, 2, 0.6]	6115	9.4
[0.054, 2, 0.4]	6757	9.4
[0.074, 2, 0]	6848	9.1

Example cases shown for each value of SD σ_app_ where there was the least number of active cells (deterministic networks), and for the five cases where there were the least number of active cells (noisy networks).

Thus, these model outputs suggested that an appropriate balance between spike frequency adaptation and excitatory connectivity in CA1 PYR cell networks could provide an essential mechanism for theta population bursts. However, the majority of PYR cells in the models were active during the population bursts, which is not consistent with experiment. This led us to consider how inhibitory cells may contribute to burst dynamics.

#### Excitatory-inhibitory (PYR-PV^+^ cell) networks, deterministic

To build networks with both excitatory and inhibitory cells, we first took advantage of previous work in which cellular models for PV^+^ fast-spiking cells were developed based on experimental recordings from the whole hippocampus preparation (Ferguson et al., 2013). Given this work, estimates of EPSCs onto PV^+^ cells of ∼1000 pA, and that we want PV^+^ cells to fire coherent bursts, we set *g_pv_* = 3 nS (Ferguson et al., 2013; Skinner and Ferguson 2013).

Next, rather than setting the excitatory drive to PV^+^ cell network as deterministic “other” input as has been done previously in Ferguson et al., (2013) (see middle schematic of Fig. 2), we created networks in which the excitatory drive comes directly from the 10,000 PYR cell network. This is shown in the right schematic of Fig. 2. Because our model is designed to explore oscillatory activity intrinsic to the CA1 region of the hippocampus, input from other regions is not specifically included. We chose example PYR cell networks with distinct firing patterns (nonfiring, stable bursts, etc.) and explored how the connectivity between PYR cells and PV^+^ cells affects network activity (Ferguson, 2015). This limited set of deterministic, excitatory-inhibitory network simulations provided a motivating basis for the expanded set of simulations presented in Results.

In PYR cell networks that exhibit stable bursts, introducing PV^+^ cells does not ensure that the bursts are maintained, but instead requires that the connection probability from PV^+^ to PYR cells (*c_PV_*_,_*_PY__R_*) surpasses a critical value. This critical connectivity value depended on the PYR-PV^+^ connection probability (*c_PY__R_*_,_*_PV_*_)_, as it could not be drastically lower than this critical value. Interestingly, if stable bursts are maintained, the frequency of the population bursts is always higher in the PYR-PV^+^ cell networks relative to the PYR cell networks alone. We note that for each set of simulations, we did not alter the excitatory drive to the PYR cells, and thus the cells did not increase their firing owing to an external change in the amount of excitation. Rather, this increased burst frequency is due to the postinhibitory rebound spiking of the PYR cells as a response to the inhibitory input from the PV^+^ cells.

**Figure 4.**
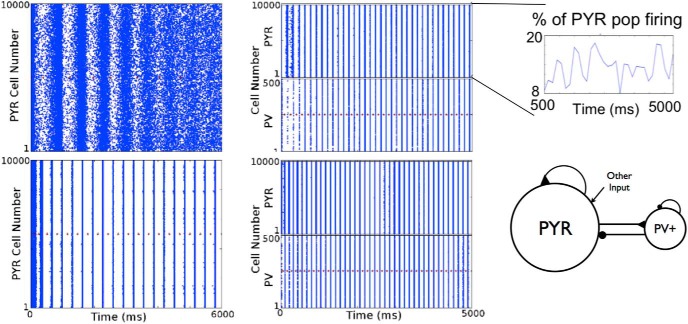
Deterministic excitatory-inhibitory PYR-PV^+^ cell networks. Examples of population burst stabilization, sparse excitatory firing, and network frequency increase are shown. PYR cell networks are shown on the left (same examples from Fig. 3). PYR-PV^+^ cell networks are shown on the right with connectivity parameters *c_PY__R_*_,_*_PV_*_,_ *c_PV_*_,_*_PY__R_* of (0.05,0.2) and (0.01, 0.2) for top and bottom, respectively. Inset shows that PYR cells firing is <20%. The population burst frequency increases from 3.1 to 6.7 Hz (bottom).

Alternatively, if the original PYR cell networks were unstable or nonfiring, stable population bursts in the theta frequency range could emerge with the inclusion of the PV^+^ cell population. In all cases, and in contrast with our PYR cell networks, it was possible to simultaneously obtain theta frequency population bursts and sparse firing of the PYR cells. These observations are illustrated in Fig. 4. Thus, even when oscillations did not exist or were not stable in the PYR cell networks, the influence of PV^+^ cells could lead to stable network rhythm generation and sparse firing. However, this depended on an appropriate balance of connection probabilities between the two populations.

Overall, we found that PYR cell networks could exhibit coherent firing at various frequencies (≈0.5–6 Hz), but when theta frequencies were produced, essentially all PYR cells were recruited to fire in every cycle, a behavior that is not consistent with what is seen in the experimental setting, where PYR cells sparsely fire. However, the inclusion of PV^+^ cells allowed sparse firing to emerge while maintaining theta bursts, but of increased frequency, or generating theta bursts. Because we did not change excitation in any other way, a key contributor to this is postinhibitory rebound firing. Thus, population bursts at theta frequency in the PYR-PV^+^ cell networks with sparse firing depend on a number of factors, including the amount of input that the cells receive at any given point in time. As such, the precise connection probabilities in the models is not the essence, but rather the relative balance between excitation and inhibition. More complete exploration and analyses are needed to untangle this.

## Results

We use network models that are minimal but at the same time are constrained by experiment. In this way, we reduce the uncertainty in choices for parameters and parameter values. Detailed reasoning and rationale for our choices are provided in Materials and Methods. We examine whether it is possible to capture the experimental observations, and if so, what are the underlying mechanisms that allow this? To start to address this, we have presented previous work and motivating modeling simulations using deterministic networks. Although limited, these simulations showed that there is an intricate tangling of cellular properties and excitatory and inhibitory balances that underlie the generation of theta population bursts with sparse PYR cell firing in the whole hippocampus preparation. That is, there are many interconnected factors. We thus performed an expanded set of simulations using more biologically realistic input to obtain insight into possible underlying mechanisms.

### Noisy networks

#### Excitatory PYR cell networks

We performed simulations with noisy, excitatory input. That is, “other” input in Fig. 2 is given by a stochastic rather than a deterministic process (see Materials and Methods). Because we know that PYR cells do not receive large EPSCs during the endogenous theta rhythm (Huh et al., 2016), we can focus on small mean excitatory conductances. That is, an EPSC of 20 pA into a PYR cell would give an excitatory conductance estimate of <1 nS, given excitatory reversal potential and resting voltage values. Given this, and that PYR cell recurrent connectivity is minimal, we consider excitatory drive (*g_e_*_,_*_mean_*) values of 0–2 nS to fully encompass the biological situation in the whole hippocampus preparation.

**Figure 5.**
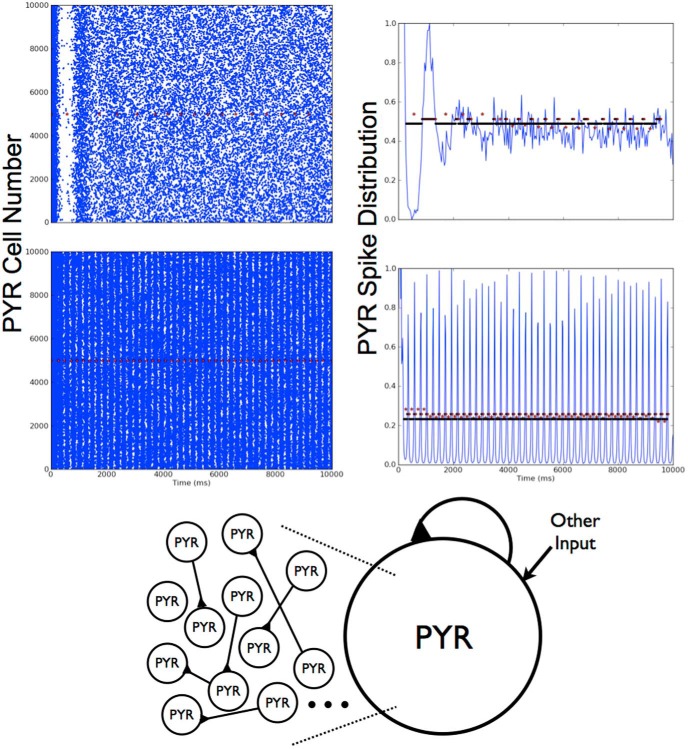
Noisy excitatory PYR cell networks. Two examples in which theta rhythms (population bursts) are either present (bottom) or not (top). On the left is the raster plot, and on the right is the normalized spike distribution. Red stars delineate the automatic detection of separate bursts. Parameter values: *g_e_*_,_*_mean_* = 0, *g_pyr_* = 0.074, σ*_e_* = 0.2 (top row: no theta rhythm); *g_e_*_,_*_mean_* = 1, *g_pyr_* = 0.074, σ*_e_* = 0.2 (bottom row: 4.4 Hz rhythm).

As would be expected, the patterns in these noisy networks are more variable than the deterministic PYR cell network simulations. However, similar to the deterministic simulations, burst frequency increased with increasing excitatory drive (*g_e_*_,_*_mean_*) and decreased with increasing recurrent synaptic strength (*g_pyr_*). There were no theta frequency bursts for *g_e_*_,_*_mean_* = 0, but for larger excitatory drives (*g_e_*_,_*_mean_* = 1 or 2), there were theta frequency population bursts except for one case that was outside of the theta frequency range: *g_pyr_* = 0.094 nS, *g_e_*_,_*_mean_* = 1, σ*_e_* = 0.6. Example output is shown in Fig. 5 with (bottom) or without (top) population bursts. Also shown in Fig. 5 is the PYR cell spike distribution that was used to determine whether bursts were present (see Materials and Methods).

The number of active PYR cells always exceeded 50%. Table 3 provides details of the five networks with the most sparse PYR cell firing. For weakly adapting cells, no theta rhythms were obtained for *g_e_*_,_*_mean_* = 0 or 1 but could emerge for larger input values. Because this would be beyond EPSCs values observed in experiments, we did not do further detailed explorations using weakly adapting cells. Thus, similar to the deterministic PYR cell network simulations, theta population bursts are present but never with sparse firing of PYR cells in more realistic, noisy PYR cell networks.

#### Excitatory-inhibitory PYR-PV^+^ cell networks

**Figure 6.**
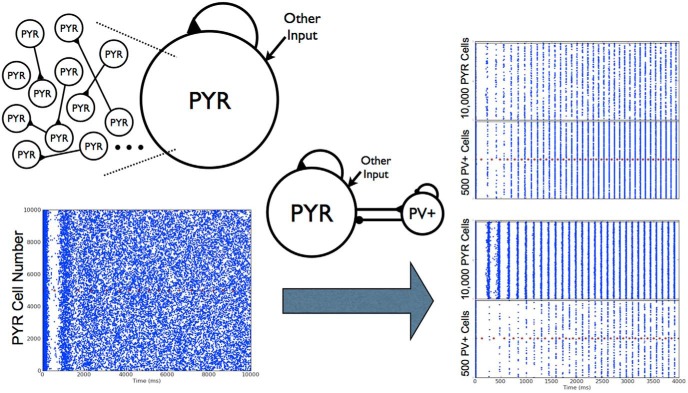
Noisy PYR to PYR-PV^+^ cell network transition. From one of the PYR cell network examples in Fig. 5 where there are no theta bursts, two examples with different connectivities of PYR-PV^+^ cell networks are shown in which theta population burst rhythms emerge. Connectivity parameters *c_PY__R_*_,_*_PV_*_,_ *c_PV_*_,_*_PY__R_* of (0.2, 0.5) and (0.02, 0.5) for top and bottom, respectively on the right. Other parameter values: *g_pyr_* (nS), *g_e_*_,_*_mean_* (nS), *g_pyr-pv_* (nS), *g_pv-pyr_* (nS) = [0.074, 0, 0.2, 3, 8.7].

**Figure 7.**
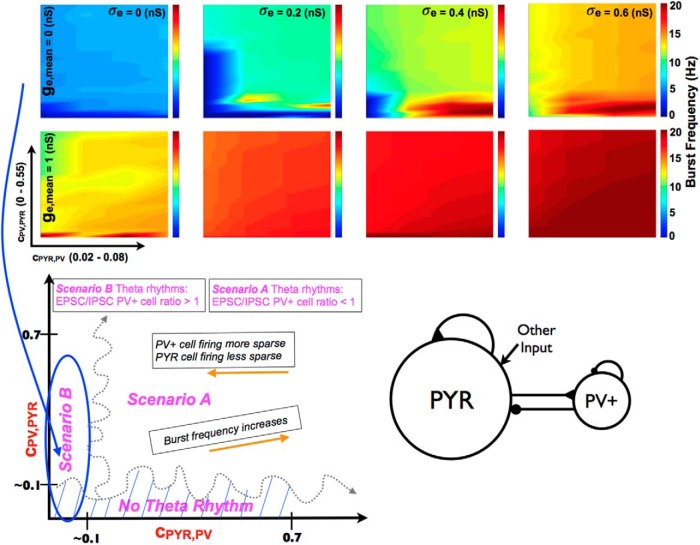
Parameter balances for the existence of theta rhythms in PYR-PV^+^ cell networks. Top: Burst frequency summarized for a range of parameter values. Color plots shows how burst frequency changes for a range of input to the PYR cell population and for a range of connectivities between PYR and PV^+^ cell populations. Note that *c_PYR_*_,_*_PV_* is shown for a much smaller range than *c_PV_*_,_*_PYR_*. Moving horizontally, one sees that increasing σ*_e_* typically leads to an increase in burst frequency. It is also clear that beyond *g_e_*_,_*_mean_* = 0, theta frequency rhythms are not present if σ*_e_* is nonzero. Other parameter values: *g_pyr_* (nS), *g_pyr-pv_* (nS), *g_pv-pyr_* (nS) = [0.014, 3, 8.7]. Bottom: Theta rhythm generation overview. This schematic summarizes the balances in the generation of theta rhythms and their characteristics in the network models. Two scenarios, A and B, can be identified based on connectivity balances (*c_PV_*_,_*_PYR_* and *c_PYR_*_,_*_PV_*_)_ and are approximately delineated by the squiggly gray dashed lines. Note that these separations are illustrative, as the exact connectivity boundary value will depend on the other parameters in the models. However, it is clear from the many simulations done and analyzed that one is able to differentiate these regions. Network models characteristics (frequency, and PV^+^ and PYR cell firings) are given in boxes with orange arrows. Scenarios A and B are differentiated by their EPSC/IPSC ratios to PV^+^ cells, as given in magenta text.

**Figure 8.**
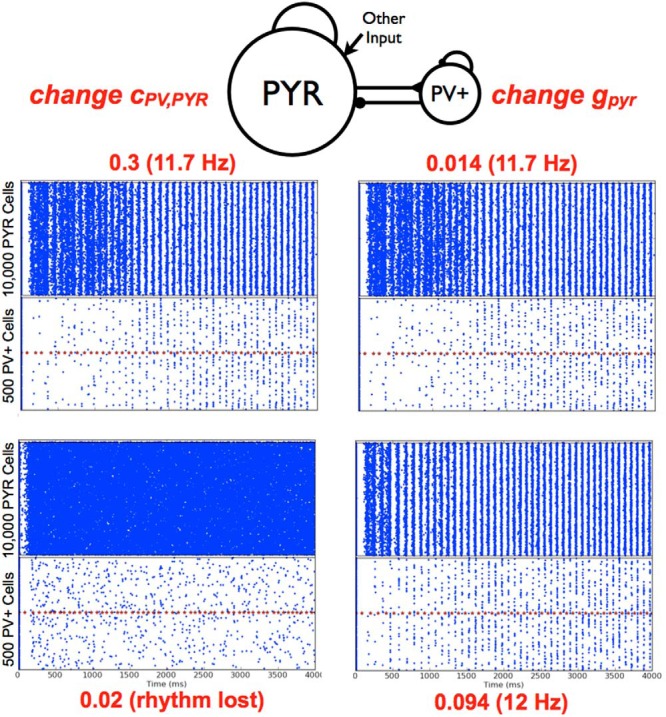
PYR-PV^+^ cell networks, parameter variations. Left: Example raster plots for decreasing *c_PV_*_,_*_PYR_*. Two examples are shown, with the *c_PV_*_,_*_PYR_* value shown in red along with the network frequency as appropriate. The average number of cells firing per burst are 548 (PYR cells) and 22 (PV^+^ cells) for *c_PV_*_,_*_PYR_* = 0.3. Other parameter values: *g_pyr_*, *g_e_*_,_*_mean_*, *g_pyr-pv_*, *g_pv-pyr_*, *c_PYR,PV_* = [0.014, 0, 0.6, 3, 8.7, 0.02]. Right: Example raster plots for changing *g_pyr_*. Two examples are shown, with the *g_pyr_* value shown in red along with the network frequency. The first, top example is the same as the one on its left. The average number of cells firing per burst are 514 (PYR cells) and 34 (PV^+^ cells) for *g_pyr_* = 0.094 nS. Other parameter values: *g_e_*_,_*_mean_*, σ*_e_*, *g_pyr-pv_*, *g_pv-pyr_*, *c_PYR,PVr_*, *c_PV,PYR_* = [0, 0.6, 3, 8.7, 0.02, 0.3].

A full exploration of connectivities (*c_PV_*_,_*_PY__R_* and *c_PY__R_*_,_*_PV_*_)_ with *g_pyr_* = 0.014 and *g_e_*_,_*_mean_* = 0, 1, and 2 nS was done. We first note that, similar to the deterministic simulations, theta bursts could be present in PYR-PV^+^ cell networks even if PYR cell networks did not have any population bursts. An example of this is shown in Fig. 6. A summary of the burst frequencies is shown in the top part of Fig. 7 for two excitatory drives (*g_e_*_,_*_mean_* = 0 and 1 nS). Theta frequency bursts encompass colors that range from light blue to orange. Given this, it is clear that PYR-PV^+^ cell networks with *g_e_*_,_*_mean_* = 1 nS had burst frequencies that exceeded theta, except for when σ*_e_* = 0 nS, meaning noiseless networks. For *g_e_*_,_*_mean_* = 2 nS, the burst frequencies far exceeded theta frequencies (not shown). Also, since larger *g_pyr_* values result in an increased burst frequency (see Fig. 8, right), it did not make sense to do additional simulations with *g_e_*_,_*_mean_* = 1 or 2 nS. This thus led to a focus on simulations with *g_e_*_,_*_mean_* = 0 nS. The full range of *g_pyr_* values were simulated along with explorations of PYR to PV^+^ and PV^+^ to PYR cell conductance values (see Materials and Methods).

From a computational analysis that consisted of several thousands of simulations, we were able to obtain a lay of the land in terms of required parameter balances for theta rhythms to occur, as well as their characteristics. This is schematized in the bottom of Fig. 7, where the connectivity ranges that refer to the summarized plots above are indicated by the blue ellipse. As can be seen, they are summarized only for smaller *c_PY__R_*_,_*_PV_* values. However, as *c_PY__R_*_,_*_PV_* increases, the burst frequency increases further (not shown). As noticed in the motivating deterministic simulations, and similarly here for the noisy runs, if *c_PV_*_,_*_PY__R_* is too small, theta bursts are not present. That is, there needs to be enough connectivity from PV^+^ to PYR cells to have postinhibitory rebound firing of an appropriate amount in PYR cells for theta bursts to occur. An example of how theta bursts are lost (when *c_PY__R_*_,_*_PV_* is also small) is shown in Fig. 8, left.

### An essence of theta rhythm generation and experimental data matching

From our noisy and motivating deterministic simulations, we are able to distinguish two scenarios, A and B, by which theta rhythms occur. It is because of the wide swaths of simulations and analyses of them that we are able to identify these different scenarios as shown in Fig. 7. As schematized, the transition between scenarios is not meant to be literal but illustrative, to consider the several other parameters that would affect the exact transitions. The difference between these two scenarios lies in how active the PYR and PV^+^ cell populations are, which in turn affects the EPSCs and IPSCs received by them.

**Figure 9.**
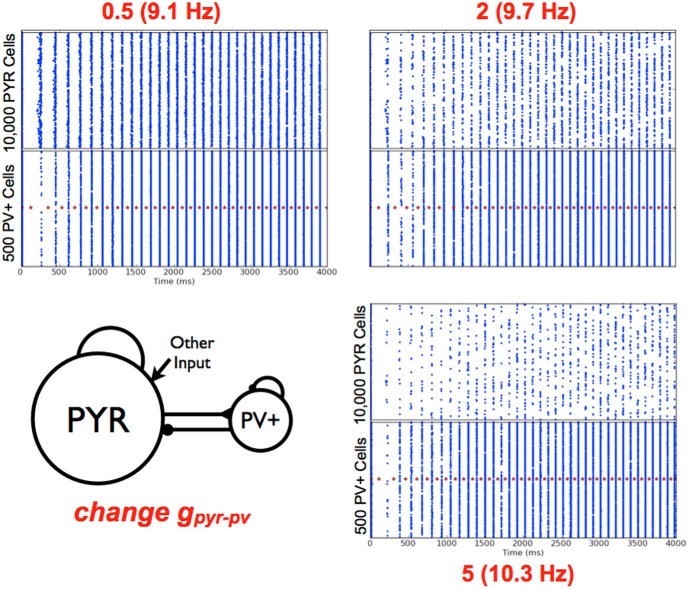
Changing excitatory conductance *g_pyr_*_-_*_pv_* from PYR to PV^+^ cells. Three example raster plots are shown, with the *g_pyr_*_-_*_pv_* value shown in red with the network frequency as appropriate. The average number of cells per population burst is 290 (PYR) and 343 (PV^+^) for *g_pyr_*_-_*_pv_* = 0.5 nS; 81 (PYR) and 288 (PV^+^) for *g_pyr_*_-_*_pv_* = 2 nS; 32 (PYR) and 238 (PV^+^) for *g_pyr_*_-_*_pv_* = 5 nS. Other parameter values: *g_e_*_,_*_mean_*, σ*_e_*, *g_pyr_*, *g_pv-pyr_*, *c_PYR,PV_*, *c_PV,PYR_* = [0, 0.2, 0.084, 6, 0.4, 0.5].

So how do theta frequency bursts emerge in PYR-PV^+^ cell networks? We first point out that *g_e_*_,_*_mean_* = 0 nS with nonzero σ*_e_* is the appropriate “other” input to use to have theta frequency population bursts. This is because of results from PYR cell networks and knowledge of EPSC values to PYR cells in experiments. Also, as noted above, if *c_PV_*_,_*_PY__R_* is not large enough, there will be no theta rhythms (Fig. 8, left). This immediately indicates the importance of postinhibitory rebound spiking in PYR cells to generate theta bursts. However, while *c_PV_*_,_*_PYR_* cannot be zero (since this would mean that PV^+^ cells are not coupled to PYR cells and one would have PYR cell network output, which we know has no theta bursts for *g_e_*_,_*_mean_* = 0), the exact nonzero value needed for theta bursts to emerge also depends on *c_PYR_*_,_*_PV_*. The example shown in Fig. 8 (left) is for a small value of *c_PYR_*_,_*_PV_*, and the PV^+^ cells are not able to fire much or coherently. If *c_PV_*_,_*_PYR_* is decreased beyond a critical value, the PYR-PV^+^ cell networks do not generate theta frequency bursts, as the PYR cells do not exhibit enough postinhibitory rebound to enable robust burst firing in the theta range. The model results are consistent with previous experimental findings from Goutagny et al., (2009), suggesting the importance of postinhibitory rebound firing in the generation of theta rhythms. Once one is within parameter balance regimes with robust theta frequency bursts, the frequency increases with increasing *c_PYR_*_,_*_PV_* and less so for increasing *c_PV_*_,_*_PYR_*, as determined from an overall examination of the simulations. This observation is particularly apparent in the upper summary plot of Fig. 7 (*g_e_*_,_*_mean_* = 0 and σ*_e_* = 0.6). We illustrate this with a slanted box (burst frequency increases) in the schematic of Fig. 7. The changing burst frequency can be seen if *g_pyr_*_-_*_pv_* rather than *c_PYR_*_,_*_PV_* is modified, as shown in Fig. 9.

The particular parameter balances that allow the generation of theta population bursts affect not only the specific frequency of the population burst but also how robust it is, that is, how easily discernible it is (see Materials and Methods). This interdependence of cellular and synaptic properties affects how much the PYR and PV^+^ cell populations fire. It is clear that the addition of the PV^+^ cell population is what allows the PYR cell population to fire sparsely. However, how sparse the firing of PYR and PV^+^ cells are depends on where the parameter balance lies. From our simulations, we observe that as *c_PYR_*_,_*_PV_* decreases, the firing of PV^+^ cells becomes more sparse and PYR cells become less sparse within a theta population burst. This is illustrated by the box in the Fig. 7 bottom schematic. It makes sense that PV^+^ cells would fire less, as they are receiving less excitatory drive with a reduced *c_PYR_*_,_*_PV_*. However, that PYR cells would fire less sparsely when the PV^+^ cells are firing less indicates that how much the PYR cells fire is not only dependent on postinhibitory rebound firing. There are clearly different balances going on. It is these different balances as brought forth from our thousands of simulations and analyses that allowed us realize that one could distinguish two scenarios by which theta rhythms emerge. Specifically, in scenario A, the PV^+^ cells fire less sparsely and the PYR cells fire more sparsely than in scenario B.

**Figure 10.**
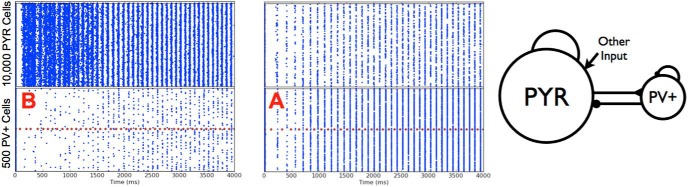
Theta rhythm generation by the two different scenarios. Left: Scenario B, same example as in Fig. 8 top. Right: Scenario A, the average number of cells per population burst is 86 (PYR) and 185 (PV^+^). Parameter values: *g_pyr_*, *g_e_*_,_*_mean_*, σ*_e_*, *g_pyr_*_-_*_pv_*, *g_pv-pyr_*, *c_PV,PYR_*, *c_PYR,PV_* = [0.084, 0, 0.2, 3, 8.7, 0.5, 0.2].

In Table 4, we present analyses regarding the average number of cells firing per burst as well as the average number of spikes per cell per burst for several parameter sets. From this table, the two different scenarios described above regarding the relative firing of PYR and PV^+^ cells can be appreciated in more detail. The third and fourth columns of Table 4 show examples where the proportion of actively firing PYR cells are larger or smaller, and similarly for PV^+^ cells. That is, it appears that a relative amount of sparseness can be distinguished. In scenario B, the PYR cells are less sparse and are less tightly bound to fire phase-locked with PV^+^ cells due to the postinhibitory rebound, and the PV^+^ cell firing is more sparse. By contrast, in scenario A, the PV^+^ cells fire more, and postinhibitory rebound plays a stronger role to more tightly control PYR cell phase-locking (so more tightly lined up with PV^+^ cells). In this way, scenarios A and B can be differentiated by how much of a role postinhibitory rebound plays in the subsequent theta rhythm. These differences can be seen by comparing the cases shown in Fig. 10 and examining explicit numbers as shown in Table 4. We note that although it is clear that our models are in line with experimental observations in terms of PV^+^ cells spiking more than PYR cells during theta rhythms (population bursts) in the model (compare fifth and sixth columns of Table 4), how much spiking PYR and PV^+^ cells exhibit is not in line with experiment. Specifically, model PV^+^ cells do not spike on each population burst as has been observed experimentally.

**Table 4.** PYR-PV^^+^^ cell network scenarios, firings

Parameter **g*_*pyr*_* (nS), σ*_*e*_* (nS), **g*_*pyr-pv*_* (nS), **g*_*pv-pyr*_* (nS), **c*_*PYR,PV*_*, **c*_*PV,PYR*_*, **g*_*e*_*_,_*_*mean*_* = 0 nS	Burst frequency (Hz) (from fft)	Average number of active PYR cells (/10,000) per burst	Average number of active PV^+^ cells (/500) per burst	Average number of spikes (/PYR cells) per 100 bursts	Average number of spikes (/PV^+^ cell) per 100 bursts
= [0.084, 0.2, 3, 8.7, 0.4, 0.5]	10	53	273	1	55
= [0.084, 0.2, 3, 8.7, 0.2, 0.5]	9.7	86	185	1	37
= [0.084, 0.2, 3, 8.7, 0.04, 0.5]	8.9	300	79	3	16
= [0.084, 0.2, 3, 8.7, 0.02, 0.5]	8.3	522	54	5	11
= [0.084, 0.2, 3, 8.7, 0.4, 0.3]	10	53	249	1	50
= [0.084, 0.2, 3, 8.7, 0.4, 0.7]	10	54	280	1	56
= [0.014, 0.6, 3, 8.7, 0.02, 0.3]	11.7	548	22	2	8
= [0.094, 0.6, 3, 8.7, 0.02, 0.3]	12	514	34	5	7
= [0.084, 0.2, 0.5, 6, 0.4, 0.5]	9.1	290	343	3	69
= [0.084, 0.2, 2, 6, 0.4, 0.5]	9.7	81	288	1	58
= [0.084, 0.2, 5, 6, 0.4, 0.5]	10.3	32	238	<.5	48

Examples to illustrate the relative firing of PYR and PV^+^ cell populations leading to a subsequent delineation of scenarios in Table 5.

Excitatory and inhibitory currents for the chosen parameter sets in Table 4 are shown in Table 5. Specifically, EPSCs and IPSCs to PYR and PV^+^ model cells are measured, and these values along with their ratios are given in Table 5. We note that there can be large EPSCs to PV^+^ cells and small EPSCs to PYR cells as observed experimentally. However, there is not always an appropriate match; IPSCs to PYR cells are too large and IPSCs to PV^+^ cells are too large in some cases. If current ratios rather than currents are compared, then it is always the case that EPSC/IPSC ratios are appropriate for PYR cells relative to experiments, but only in some cases are the EPSC/IPSC ratios for PV^+^ cells somewhat appropriate, that is, close to or greater than 1. As such, we find that the EPSC/IPSC ratio to PV^+^ cells, but not the EPSC/IPSC ratio to PYR cells, allows us to distinguish between scenarios A and B. We then conclude that scenario B, but not scenario A, is consistent with the experimental data, and so is the situation that is appropriate for the biological system; that is, one in which postinhibitory rebound, although required to be present, plays less of a role in theta rhythm generation.

**Table 5.** PYR-PV^^+^^ cell network scenarios, currents

Parameter **g*_*pyr*_* (nS), **σ*_*e*_* (nS), **g*_*pyr*_*_-_*_*pv*_* (nS), **g*_*pv*_*_-_*_*pyr*_* (nS), **c*_*PYR*_*_,_*_*PV*_*_,_ **c*_*PV*_*_,_*_*PYR*_*, **g*_*e*_*_,_*_*mean*_* = 0 nS	EPSC to PYR cell (pA)	IPSC to PYR cell (pA)	E/I ratio (PYR cell)	EPSC to PV^+^ cell (pA)	IPSC to PV^+^ cell (pA)	E/I ratio (PV^+^ cell)	Scenario
= [0.084, 0.2, 3, 8.7, 0.4, 0.5]	4	1500	≪ 1	700	1800	< 1	A
= [0.084, 0.2, 3, 8.7, 0.2, 0.5]	4	1600	≪ 1	550	1300	< 1	A
= [0.084, 0.2, 3, 8.7, 0.04, 0.5]	5	1500	≪ 1	350	550	≈ 1	B
= [0.084, 0.2, 3, 8.7, 0.02, 0.5]	7	730	≪ 1	300	275	≈ 1	B
= [0.084, 0.2, 3, 8.7, 0.4, 0.3]	4	2500	≪ 1	650	1950	< 1	A
= [0.084, 0.2, 3, 8.7, 0.4, 0.7]	4	1150	≪ 1	740	1770	< 1	A
= [0.014, 0.6, 3, 8.7, 0.02, 0.3]	1	410	≪ 1	340	200	> 1	B
= [0.094, 0.6, 3, 8.7, 0.02, 0.3]	7	430	≪ 1	220	200	≈ 1	B
= [0.084, 0.2, 0.5, 6, 0.4, 0.5]	7	2000	≪ 1	480	2450	< 1	A
= [0.084, 0.2, 2, 6, 0.4, 0.5]	4	2200	≪ 1	650	2000	< 1	A
= [0.084, 0.2, 5, 6, 0.4, 0.5]	4	2700	≪ 1	870	1900	< 1	A

The same examples as in Table 4 but now showing excitatory and inhibitory currents and the delineated scenarios

## Discussion

### Summary, theta essence, explanation, and predictions

We have developed microcircuit models and obtained an explanation for how theta rhythms can be generated in the hippocampus. We used a strategy, as schematized in Fig. 1, in which we took advantage of an experimental context of an intrinsic CA1 theta with developed mathematical models, leveraged theoretical studies, and did extensive parameter variation analyses. This computational analysis allowed us to differentiate between two scenarios of how theta rhythms could be generated, and only one of them is consistent with the experimental data. We suggest that spike frequency adaptation and postinhibitory rebound in CA1 pyramidal cells are sufficient conditions (building blocks) for the generation of theta rhythms with sparse excitatory cell firing. Moreover, if it is the case that spike frequency adaptation is required, then it is necessary to have postinhibitory rebound. Further, our network simulations predict that theta rhythms are present when the input to the PYR cells has a nonzero fluctuating input conductance of 0.6 nS or less and a zero mean conductance. Our network models are minimal but were able to capture an essence of the experimental data. As such, we consider our models a foundation on which to build.

This building block understanding leads to the following claims and predictions. We first note that if PV^+^ cells are removed from the network, then there are no spontaneously generated theta rhythms. This is in accordance with Amilhon et al. (2015), in which the optogenetic silencing of PV^+^ cells abolished intrinsically generated theta rhythms in a whole hippocampus preparation *in vitro*. We predict that spike frequency adaptation in PYR cells is required for theta rhythms to be present, as balanced by a large enough PYR cell network with connectivity that is minimal. The amount of spike frequency adaptation controls the existence of theta rhythms and its resulting frequency, as built on our understanding from the theoretical mechanism. Thus, if cellular adaptation in PYR cells is selectively adjusted—say, by modulating potassium and calcium-activated potassium channels—then one should see an effect on the frequency of theta rhythms. If cellular adaptation is reduced enough, there would be no theta rhythms. Further, by selectively altering either the amount of connectivity or the conductance from PYR to PV^+^ cells, the theta frequency would be strongly affected. Alternatively, if the amount of connectivity or the conductance from PV^+^ to PYR cells is selectively adjusted, then the theta frequency would be less affected. Too much reduction, however, would result in no theta rhythms, as postinhibitory rebound would not be present. Finally, we explored a wide range of parameter values in our network models, but only a portion of them are in agreement with the experimental data regarding EPSC/IPSC ratios. As such, we predict that theta rhythms in the hippocampus are generated via scenario B, where the EPSC/IPSC ratio to PV^+^ cells is greater than 1 (Fig. 7). Although postinhibitory rebound is still an important element to have theta rhythms (i.e., *c_PV_*_,_*_PYR_* cannot be zero) in scenario B, it plays less of a role relative to scenario A. If scenario B is the mechanism underlying theta rhythms in the hippocampus, we predict that the probability of connections or conductance from PV^+^ to PYR cells is larger than the probability of connection or conductance from PYR to PV^+^ cells (see Fig. 7 schematic). It is of interest to note that large EPSCs onto PV^+^ cells can be present even if there is a minimally connected CA1 PYR cell network that sparsely fires. This is necessarily due to the large size of the PYR cell network.

Our explanation here of theta generation in the hippocampus has Aristotelian elements of efficient, material, and formal causes (Falcon, 2015). That is, we find that spike frequency adaptation and postinhibitory rebound in large, minimally connected networks (efficient cause) consisting of PYR and PV^+^ cells connected by inhibitory (*GABA_A_*) and excitatory (*AMPA*) synapses (material cause) generates theta rhythms. The models require parameter balances of cellular adaptation in a large PYR cell network with enough connectivity and postinhibitory rebound due to PV^+^ cells, and larger PV^+^ to PYR than PYR to PV^+^ cell connectivity (formal cause). We note that a final cause cannot be considered with the present model, as it uses a whole hippocampus preparation *in vitro*, so function cannot be directly addressed. There are several ongoing efforts to consider theta rhythm functions (Colgin, 2013), but as emphasized by Colgin (2013), it is important to know what the underlying causes of theta generation are in the first place. In future work, for example, one might consider how our modeling work could be expanded and linked to functional theta modeling studies such as recent efforts by Chadwick et al. (2016) to capture the flexibility of theta sequences by including phase-precessing interneurons in septo-hippocampal circuitry.

It is important to emphasize that the form of theta modeled and explained here is one that is intrinsic to the hippocampus. However, with a baseline model of how the intrinsic CA1 microcircuit can produce theta rhythms in the absence of oscillatory synaptic input, we could gain insight into how external sources influence and build on this CA1 microcircuit. There is always the possibility that in some cases the microcircuit is just perturbed and the mechanisms remain intact, but in other cases it could be that the mechanisms are entirely different. Our models may help to identify which is the case, as it makes clear testable predictions as described above.

### Related modeling studies

Previous mathematical modeling studies of theta rhythms have been described (Kopell et al., 2010; Ferguson and Skinner, 2015). Earlier models put forth theta generation mechanism ideas based on coherence between theta frequency firing in oriens-lacunosum/moleculare (O-LM) interneurons, in which a hyperpolarization-activated inward current (h-current) is critical for the coherence (Rotstein et al., 2005; Wulff et al., 2009). However, this has subsequently been shown to be unlikely, since O-LM cells do not operate as theta pacemakers (Kispersky et al., 2012), an assumption in the earlier models. In another hippocampal modeling study, it was shown that theta rhythms could be generated in a network of basket, O-LM, and pyramidal cells (Neymotin et al., 2011). Although the focus of that study was not on theta generation per se, O-LM cells were strongly implicated in contributing to theta rhythms.

Based on recordings from the hippocampus of behaving rats, models were developed to support the observations that h-currents in pyramidal cells were needed to allow theta frequency spike resonance to occur (Stark et al., 2013). The mathematical models were focused on considering the contribution of h-currents in pyramidal cells and did not specifically consider connectivity between PYR and PV^+^ interneurons or the numbers of cells. The study emphasized the importance of postinhibitory rebound in theta rhythms. Interestingly, this same study found that adaptation was not an important contributing factor. In our modeling study, both cellular adaptation and postinhibitory rebound are needed to bring about theta rhythms in the hippocampus. However, unlike Stark et al. (2013), which had an experimental *in vivo* context, our models are based on an intrinsically generated hippocampal theta rhythm, thus removing contributions from other brain structures. In this way, we were able to focus on whether and how excitatory-inhibitory networks could produce theta rhythms considering network size, connectivity, and cellular characteristics. Given the complexity of theta rhythms, one would expect that there could be different balances of building blocks (cellular adaptation and postinhibitory rebound), as well as additional ones underlying theta rhythms *in vivo*. However, it is considerably more challenging to dissect out an understanding of theta rhythm generation *in vivo*. Using our network models and mechanistic understandings derived from them, this challenge could be reduced.

A full-scale biologically detailed CA1 hippocampal model has recently been developed (Bezaire and Soltesz, 2013; Bezaire et al., 2016). It is loosely based on the whole hippocampus preparation and includes eight inhibitory cell types. This model exhibits theta rhythms phase-locked with gamma oscillations and shows distinct phase relationships for different cell types. From model perturbations, interneuronal diversity, and more specifically, parvalbumin-expressing interneurons and neurogliaform cells, were found to be important in theta generation. Further, although not a particular focus, the generation of theta rhythms in these detailed network models required a particular balance of excitation. Considering Aristotle’s four causes (Falcon, 2015), this descriptive understanding of theta generation can be considered as a material cause with elements of an efficient cause. However, due to its very detailed nature, it would be difficult to acquire a formal cause from it.

In a recent study Giovannini et al. (2017) focused on the contribution of a nonspecific cation current in pyramidal cells as critical for the maintenance of theta oscillations in the isolated hippocampus preparation. Interestingly, similar to our work here, they showed that when pyramidal cells were coupled with inhibitory cells, theta oscillations became more robust. This work could be considered as a particular material cause explanation of theta rhythm generation.

### Spike frequency adaptation and postinhibitory rebound

Spike frequency adaptation as a mechanism to generate populations bursts has been used before, and previous work examined whether the amount of cellular adaptation expressed in pyramidal cells was appropriate to generate population bursts in pyramidal cell networks (Dur-E-Ahmad et al., 2011; Ferguson et al., 2015a). How best to model and examine adaptation naturally depends on the questions being considered. For example, Benda et al. (2010) examined how adaptation in the signal transmission of sensory systems can be due to either adaptation currents or dynamic thresholds. Coupled oscillator theory was used to examine how adaptation contributed to synchronization (Crook et al., 1998). In another study, a fractional leaky integrate-and-fire model to capture spike frequency adaptation was developed to set a framework to help understand information integration in neocortex (Teka et al., 2014). For our CA1 pyramidal cell models, we used an Izhikevich-type cellular model (Izhikevich, 2006), and adaptation was captured in the *d* and *a* parameters when fitted to experimental frequency–current curves (Ferguson et al., 2015b). Further, for postinhibitory rebound to be present in these models, the *b* parameter needed to be positive. Although the experimental data indicated that both strongly and weakly adapting pyramidal cells exist, this does not of course mean that there are simply two types, as the existence and amount of adaptation depends on the complement of biophysical ion channels in the cells and what is uncovered by the particular experimental protocol. In the work here, we used both types of pyramidal cell models, but networks of weakly adapting cells only were not able to produce theta rhythms given other constraints of the experimental context.

In the intact hippocampus *in vitro* preparation, CA1 pyramidal cells exhibit postinhibitory rebound inhibition (Goutagny et al., 2009; Ferguson et al., 2015b). H-currents in pyramidal cells clearly play a key role in their ability to express postinhibitory rebound. However, both the presence and the distribution of these currents, together with the distribution of other currents, need to be taken into consideration. Specifically, it was shown that postinhibitory rebound is rarely observed in physiologic conditions unless unmasked by the blocking of A-type potassium currents (Ascoli et al., 2010), and rebound and other properties vary along the longitudinal axis of the hippocampus (Malik et al., 2016). Both h-currents and A-type potassium currents are known to have a nonuniform distribution along pyramidal cell dendritic arbors, and putative functional contributions of this to temporal synchrony have been made (Vaidya and Johnston, 2013). It is interesting to note that a difference in the dorsal to ventral patterning of h-currents exists (Dougherty et al., 2013), bringing to light another possibility of rhythm modulation. Further, traveling theta waves have been observed in both rodent (Lubenov and Siapas, 2009; Patel et al., 2012) and human (Zhang and Jacobs, 2015) hippocampus, suggesting a coupled oscillator organizational motif in the hippocampus. Although a “weakly coupled oscillator” terminology has been invoked in describing these waves (Colgin, 2013), this should not be confused with the mathematical theory where the assumption of weakly coupled oscillators is used to reduce the system to a phase-coupled system that is easier to analyze (Schwemmer and Lewis, 2012).

## Limitations

Given our highly simplified and minimal network models, we did not expect to find a perfect match to the experimental data. However, it is important to note that given our minimal models, we were able to examine several thousand parameter sets, which in turn enabled us to explore and understand what balances might be important in bringing about theta rhythms. This balance and building block understanding can serve as a basis for how theta rhythm frequency and existence can be modulated by additional inputs from other brain regions, as well as modulation that would affect adaptation and postinhibitory rebound.

Our network models are minimal, but they were able to produce theta rhythms with sparse firing, as represented by population bursts, allowing us to suggest sufficient and necessary conditions for their generation. Although our models took into consideration network size, connectivity, and cellular characteristics in a clear experimental context, architecture was not considered. That is, connectivity used in the network models was random. This is clearly a simplification, especially considering the recent finding of motifs in pyramidal cells in the CA3 region of hippocampus (Guzman et al., 2016). However, it is a reasonable first approximation that allowed us to explore a wide expanse of connectivities.

Variability in intrinsic cell properties and mixing of weakly and strongly adapting pyramidal cells could be considered. Introducing variability immediately raises the question of how it should be done. For example, Harrison et al. (2015) quantified the heterogeneity within and between the neocortical pyramidal cell classes. They examined the class-dependent variance and covariance of electrophysiological parameters using simple models but did not focus directly on network dynamics or rhythms. Given our large explorations of conductances, connectivities, and noisy input, some variability was present in our simulations.

Further, only one type of inhibitory cell was included in our networks, that is, the fast-firing PV^+^ cell type. It is unlikely that only this one type of inhibitory cell contributes to theta rhythms, but we identified this as a good place to start given that Amilhon et al. (2015) found that they were essential. By no means do our models imply that other inhibitory cell types are unimportant. On the contrary, since our models clearly do not fully capture the experimental data (e.g., model PV^+^ cells fire too sparsely relative to experiment), aspects are clearly missing. Because PV^+^ fast-firing cells also include bistratified and axo-axonic cells, an expansion of PV^+^ cell networks along these lines could be considered (Ferguson et al., 2015c). Given the diversity of inhibitory cell types (Chamberland and Topolnik, 2012) and the different types of PV^+^ cells (Baude et al., 2007), we did not specifically try to scale the number of PV^+^ cells as we did for PYR cells; however, since we fully explored connectivity ranges between PV^+^ and PYR cells, this was in effect included. The inclusion of O-LM cells and other inhibitory cell types in the network models is important moving forward to be able to understand how they modulate theta rhythms (Leao et al., 2012; Amilhon et al., 2015; Sekulic and Skinner, 2017).

### Conclusions and Future Work

Our network models represent closed, self-consistent, accessible models that can generate theta rhythms in hippocampus CA1. We intend them as a starting point on which to build to understand the mechanisms underlying theta rhythm generation in the hippocampus. Specifically, the network model building block balances need to be fully analyzed so that a more solid formal cause of explanation can be obtained, beyond what was obtained from the computational, parameter variation analyses done here. Further, in combination with full-scale models such as that of Bezaire et al. (2016), it may be possible to obtain an understanding that can fully encompass efficient, material, and formal causes in the Aristotelian sense and could subsequently help in a final cause understanding.

Overall, our models can serve as a backbone on which other cell types as well as details of particular cell types (biophysical channels, dendrites, and spatial considerations), modulatory effects, and input from the medial septum can be incorporated. However, in doing this, it is important to note that interaction and testing with experiment should be designed accordingly, given the strategy used in developing our models (Fig. 1), and that a consideration of the different forms of theta is not lost. Moving forward, it will be important to include biophysical LFP models to allow direct comparisons between models and experiments regarding LFPs and provide further constraints. Interestingly, it has been shown that active subthreshold currents can lead to distinct resonances in the generation of LFPs (Ness et al., 2016).

## Synthesis

Reviewing Editor: Upinder Bhalla, National Center for Biological Sciences Decisions are customarily a result of the Reviewing Editor and the peer reviewers coming together and discussing their recommendations until a consensus is reached. When revisions are invited, a fact-based synthesis statement explaining their decision and outlining what is needed to prepare a revision will be listed below. The following reviewer(s) agreed to reveal their identity: Gaute Einevoll, Rishikesh Narayanan

The reviewers appreciated this modelling study on hippocampal theta rhythms.

There are two primary points that the authors must address:

1. The title, abstract and introduction should be tightened.

2. There is a substantial body of literature that the authors should factor

into their analysis and discussion.

In addition the reviewers had a number of specific suggestions for improving

the manuscript.

--------------------------

Reviewer 1:

Advances the Field (Required) This manuscript describes a comprehensive and accomplished modelling study of a hippocampal neural circuit particularly aimed at elucidating the prevalent theta rhythms in this system. In my mind this is a very important study as it not only aims to investigate generic properties of the network model, but rather tries to map the model to specific experiments. This is very challenging as it, for example, requires comprehensive investigation of the model for large sets of parameters. While the end result regarding what aspect of the circuitry is responsible for the theta oscillations maybe is not completely “crisp and clean”, progress is clearly made. A nice prediction is for example that the connection probability from PV+ to PYR cells needs to be larger than from PYR to PV+ cells for theta rhythms to emerge (and other properties be compatible with experiments as well).

Comments to the Authors (Required) This manuscript describes a comprehensive and accomplished modelling study of a hippocampal neural circuit particularly aimed at elucidating the prevalent theta rhythms in this system. In my mind this is a very important study as it not only aims to investigate generic properties of the network model, but rather tries to map the model to specific experiments. This is very challenging as it, for example, requires comprehensive investigation of the model for large sets of parameters. While the end result regarding what aspect of the circuitry is responsible for the theta oscillations maybe is not completely “crisp and clean”, progress is clearly made. A nice prediction is for example that the connection probability from PV+ to PYR cells needs to be larger than from PYR to PV+ cells for theta rhythms to emerge (and other properties be compatible with experiments as well).

The construction of a network model like this obviously involves a lot of choices regarding model elements and model parameters. In general the choices made seem sensible. I have, however, a question regarding how the synaptic input is modelled. Conductance-based synapses with short time constants for the conductivity are chosen. This is maybe for synapses placed on the somas of the cells, but what about distally placed synapses on apical dendrites of pyramidal cells?

Minor points:

- The title is a bit vague and sounds more suitable for a “perspective” or review article. Maybe it could be made more specific and sharp?

- In the Intro describing the “theta rhythm” it should be explained what it is that oscillates: LFP? EEG? membrane potentials? firing rates?

- Above Eq. 1 it is stated that a slight modification of the Izhikevich models was to “reproduce the spike width”. But I though that in the Izhikevich model the spike is added by hand as in integrate-and-fire models?

- Table 2, shouldn it be 10,000 pyr cells instead of 10,0000?

- l. 171: rheobase current for strong adapting pyr, of 5 pA does not match table1 of 45 pA

- l. 237: Why is the “average membrane potential” used as a measure for the population activity? Why not the population firing rates?

- Figure 7: labels on axes of 2d colour panels are too small

- The authors may be unaware of the recent paper by Ness, Riemme & Einevoll (J Physiol 2016) where it is shown that active dendritic conductance can lead to “resonances” in the generated LFP that could be in the theta-frequency range even with a flat frequency spectrum in the synaptic input. Maybe of relevance for the Discussion?

--------------------------------

Reviewer 2:

Comment

Advances the Field (Required) Moderate advances to the field. I have provided clear details in the first and second paragraphs of my comments to the authors.

Comments to the Authors (Required)

I am positive about the manuscript, although I am not very convinced that the conceptual advances presented here are significant. I am not convinced because (i) the manuscript misses several studies from within the hippocampal oscillatory literature that also model hippocampal theta; (ii) the manuscript builds on implicit assumptions that there is only one route to the generation of theta, which is against established literature, and places too much emphasis on an in vitro experiment that results in one form of theta whose relevance to in vivo conditions has not been fully established; (iii) the presentation of the manuscript seems to implicitly assume that the theoretical bases for the generation of oscillatory rhythms in “individual neurons and small circuits” are somehow different from the building blocks for oscillation generation in the mammalian brain. However, a large body of literature spanning the mammalian hippocampal formation and the cortex has clearly shown that the theoretical bases are very similar, if not the same, except for differences in scale with reference to network oscillations. The role of PIR, push-pull inhibition, adaptation have all been employed to assess the roles of these components in mammalian cell-autonomous and network oscillations (e.g., studies from Traub-Whittington, Buzsaki-Wang, Koppell-Ermentrout, Izhikevich, White, Sejnowski). If this is considered, the roles of adaptation, post-inhibitory rebound and excitatory-inhibitory interactions to different frequencies of oscillations are very well established across the literature, leaving the overall conceptual advances presented here minimal.

Despite this, I am positive about the manuscript because of the scale of the analysis presented here, the extent of the parametric sensitivity analysis, and the specific conclusions and testable predictions they are able to draw about the in vitro form of theta in the whole hippocampal preparation. However, I request that the authors carefully consider the references presented below and recalibrate their analyses and presentation. I also believe that it might be more appropriate to have the abstract more detailed. The current version is short of details, does not emphasize the different routes to generate hippocampal theta and does not clearly state that current model is only with reference to one form of in vitro theta.

1. Introduction, discussion and missing literature:

The authors mention the following in their introduction:

“However, it is unclear what the building blocks for oscillation generation in the mammalian brain might be.”

“Although Jung and Kornmuller discovered theta rhythms almost eighty years ago in the rabbit (Colgin, 2013), how theta rhythms are generated is not yet clear.”

“it is traditionally thought that the medial septum (MS) is critical for the generation of theta since theta rhythms are disrupted when the MS is lesioned or inactivated. However, the hippocampus can exhibit theta rhythms without the MS (Goutagny et al., 2009).”

These statements need to be significantly qualified! The authors should note that the mechanisms behind the generation of different frequency of oscillations in mammalian systems have been studied in detail, both computationally and experimentally. It is also well recognized that there are several mechanisms that mediate these oscillations, with differential pharmacological sensitivities and distinct parametric sensitivities in network and single cell models. While some theta rhythm references are provided below, the many mechanisms (some overlapping) that are involved in gamma rhythm generation may be traced from these reviews (Buzsaki and Wang, Ann. Rev. Neuroscience, 2012; Wang, Physiological reviews, 2010)

With reference to theta oscillations, the presence of several different forms of hippocampal theta oscillations that are linked to different brain regions is well established (Buzsaki, Neuron, 2002; Buzsaki, Rhythms of the brain, 2006; Tatsuno, Analysis and Modeling of Coordinated Multi-neuronal Activity, 2015). It is also known that only one form of theta is dependent on the MS, with another intrahippocampal source that is consequent to interactions within the CA3 recurrent loop (Buzsaki, Neuron, 2002).

These different theta oscillations have been analyzed in recent years as well, systematically through the use of distinct experimental approaches.

References for the emergence of network theta oscillations in the hippocampus:

Computational model of carbachol-induced delta, theta, and gamma oscillations in the hippocampus

https://www.ncbi.nlm.nih.gov/pubmed/11769308

Ih tunes theta/gamma oscillations and cross-frequency coupling in an in silico CA3 model.

https://www.ncbi.nlm.nih.gov/pubmed/24204609

A model of atropine-resistant theta oscillations in rat hippocampal area CA1.

https://www.ncbi.nlm.nih.gov/pubmed/12231638

Cellular mechanisms of neuronal population oscillations in the hippocampus in vitro

https://www.ncbi.nlm.nih.gov/pubmed/15217333

The Influence of the Parameters on the Rhythm in Excitatory-Inhibitory Neural Network

2010 International Conference on Computational Intelligence and Software Engineering (CiSE)

Gamma and Theta Rhythms in Biophysical Models of Hippocampal Circuits

http://link.springer.com/chapter/10.1007/978-1-4419-0996-1_15

Theta oscillations by synaptic excitation in a neocortical circuit model

http://rspb.royalsocietypublishing.org/content/272/1558/101.short

Spike frequency adaptation and network oscillations:

Spike Frequency Adaptation Affects the Synchronization Properties of Networks of Cortical Oscillators

https://www.ncbi.nlm.nih.gov/pubmed/9573408

Spike Frequency Adaptation and Neocortical Rhythms

https://www.ncbi.nlm.nih.gov/pubmed/12163528

The Effects of Spike Frequency Adaptation and Negative Feedback on the Synchronization of Neural Oscillators

https://www.ncbi.nlm.nih.gov/pubmed/11387047

Experimental and modeling references for the emergence of theta oscillations in the medial septum:

The presence of pacemaker HCN channels identifies theta rhythmic GABAergic neurons in the medial septum.

https://www.ncbi.nlm.nih.gov/pubmed/18565991

GABAergic neurons of the medial septum lead the hippocampal network during theta activity.

https://www.ncbi.nlm.nih.gov/pubmed/19553449

How do glutamatergic and GABAergic cells contribute to synchronization in the medial septum?

https://www.ncbi.nlm.nih.gov/pubmed/16896519

Action potentials and relations to the theta rhythm of medial septal neurons in vivo.

https://www.ncbi.nlm.nih.gov/pubmed/10452212

Intracellular recordings from medial septal neurons during hippocampal theta rhythm.

https://www.ncbi.nlm.nih.gov/pubmed/9187280

The hippocamposeptal pathway generates rhythmic firing of GABAergic neurons in the medial septum and diagonal bands: an investigation using a complete septohippocampal preparation in vitro.

https://www.ncbi.nlm.nih.gov/pubmed/18400909

Phase segregation of medial septal GABAergic neurons during hippocampal theta activity.

https://www.ncbi.nlm.nih.gov/pubmed/15456820

In vivo contribution of h-channels in the septal pacemaker to theta rhythm generation.

https://www.ncbi.nlm.nih.gov/pubmed/15450094

In vivo intracellular recordings of medial septal and diagonal band of Broca neurons: relationships with theta rhythm.

https://www.ncbi.nlm.nih.gov/pubmed/7615035

A model of theta rhythm production in the septal-hippocampal system and its modulation by ascending brain stem pathways

https://www.ncbi.nlm.nih.gov/pubmed/11153716

Modeling synchronous theta activity in the medial septum: key role of local communicationsbetween different cell populations.

https://www.ncbi.nlm.nih.gov/pubmed/25904470

Modulation of septo-hippocampal θ activity by GABAA receptors: an experimental and computational approach

https://www.ncbi.nlm.nih.gov/pubmed/15183510

Pacemaker Neurons for the Theta Rhythm and Their Synchronization in the Septohippocampal Reciprocal Loop

https://www.ncbi.nlm.nih.gov/pubmed/11826054

Apart from this there are several modeling references for the emergence of cell-autonomous theta oscillations in mammalian neurons, including in the entorhinal cortex stellate cells (studies from Alonso and Hasselmo labs, for instance). The importance of postinhibitory rebound (PIR) for oscillations is very well established as the authors have pointed out, and as a theoretical and experimental concept it is not very different mammalian neurons that exhibit PIR as well (Huguenard and McCormick, Trends in Neurosciences, 2007; Cobb et al., Nature, 1995). Therefore, it is not entirely true that “it is unclear what the building blocks for oscillation generation in the mammalian brain might be”, especially with reference to the hippocampal theta oscillations. There is definitely a lot of debate about the contribution of different mechanisms to different theta rhythms and about the relative contributions of several different forms of theta to physiology and behavior, but the different “building blocks” that contribute to or mediate theta are definitely known. The references mentioned above are in no way complete, and provide only a small slice of the literature on mammalian oscillatory mechanisms, even if the focus is restricted to theta.

It might be appropriate if the authors focus on this extensive mammalian oscillatory literature in the introduction rather than employing this space for a general paragraph on experimental vs modeling approaches. Specifically, I don't see a specific need for the first paragraph of the Introduction. While it might fit into an overall review on modeling vs. experimental approaches to neuroscience, I don't see how it is directly relevant to the question that is posed in this manuscript or how it provides the motivation for the specific question that is being addressed here. The authors might want to consider the refocusing the introduction to be more appropriate to the question at hand, to the novelty their conclusions, especially in light of the references mentioned above, rather than delving into general arguments about the utility of one approach vs. the other.

The authors could mention relevant literature cited above, and specifically discuss the advances made in their study with reference to these advances made in the mammalian and non-mammalian literature.

2. Different forms of theta:

The study that the authors based their model on shows CA1 specific theta oscillation under in vitro conditions, and emerges after 10-50 mins of transfer to the recording chamber. The authors should comment on the in vivo relevance of these oscillations, especially because this emerges in the absence of the intact transmission of neurotransmitters of neuromodulators. Could this just be a slow homeostatic response that emerges only under lack of afferent activity under in vitro conditions, with minimal relevance for in vivo theta? Although this is not a question of direct relevance to the authors' study, it might be of importance to consider this in the discussion, especially from the broader perspective theta oscillations under an ethological framework. The authors should also emphasize and consider this (and the fact that there are several thetas in the hippocampus, with differing mechanisms) when they compare their conclusions of their study to an in vivo form of theta (line 579). To emphasize the solution presented here as the only possible solution to obtain theta rhythms is rather odd, especially given the several disparate mechanisms and brain regions involved in the generation of different forms of theta.

The title could also mention “in vitro” theta rhythms because the current title is misleading given the several theta rhythms that coexist in the hippocampus.

3. Post-inhibitory rebound in CA1 pyramidal neurons:

The authors have mentioned about how PIR is rarely observed in hippocampal CA1 neurons (Ascoli et al., J Neuroscience, 2010), and have pointed to significant differences between Ih expression. With specific reference to their conclusions, the authors might want to note that the rebound in ventral CA1 pyramidal neurons is larger compared that in their dorsal counterparts (Malik et al., Hippocampus, 2016).

4. Heterogeneities and class of excitability of different neurons:

The impact of heterogeneities have briefly been described. But this is very important to be neglected. See Wang and Buzsaki J Neuroscience, 1998 for the impact of heterogeneities on gamma oscillations. It is also important that the authors comment on the role of different types of excitability on the oscillations. e.g. see Tikidji-Hamburyan et al., J Neuroscience, 2015. It is well established that neurons in the hippocampus are not Class I integrators, but show hybrid characteristics (Ratte et al., Neuron, 2013; Tikidji-Hamburyan et al., J Neuroscience, 2015; Das and Narayanan, J Neuroscience, 2014; Das and Narayanan, J Physiology, 2015). The authors should at least discuss the implications for heterogeneities and class of excitability of different neurons on the specifics of their conclusions.

5. “However, the majority of PYR cells in the models were active during the population bursts, which is not consistent with experiment.”

Citations? Is Csisvari et al., J Neuroscience involving in vivo theta is also an appropriate citation for this???

--------------------------------
